# Cyclooxygenase-Derived Prostaglandin E_2_ Drives IL-1–Independent *Mycobacterium bovis* Bacille Calmette-Guérin–Triggered Skin Dendritic Cell Migration to Draining Lymph Node

**DOI:** 10.4049/jimmunol.2100981

**Published:** 2022-06-01

**Authors:** Veronika Krmeská, Juliana Bernardi Aggio, Susanne Nylén, Pryscilla Fanini Wowk, Antonio Gigliotti Rothfuchs

**Affiliations:** * Department of Microbiology, Tumor and Cell Biology, Karolinska Institutet, Stockholm, Sweden; and; † Instituto Carlos Chagas, Fundação Oswaldo Cruz, Curitiba, Brazil

## Abstract

Inoculation of *Mycobacterium bovis* Bacille Calmette-Guérin (BCG) in the skin mobilizes local dendritic cells (DC) to the draining lymph node (dLN) in a process that remains incompletely understood. In this study, a mouse model of BCG skin infection was used to investigate mechanisms of skin DC migration to dLNs. We found enhanced transcription of cyclooxygenase (COX)-2 and production of COX-derived PGE_2_ early after BCG infection in skin. Animals treated with antagonists for COX or the PGE_2_ receptors EP2 and EP4 displayed a marked reduction in the entry of skin DCs and BCG to dLNs, uncovering an important contribution of COX-derived PGE_2_ in this migration process. In addition, live BCG bacilli were needed to invoke DC migration through this COX-PGE_2_ pathway. Having previously shown that IL-1R partially regulates BCG-induced relocation of skin DCs to dLNs, we investigated whether PGE_2_ release was under control of IL-1. Interestingly, IL-1R ligands IL-1α/β were not required for early transcription of COX-2 or production of PGE_2_ in BCG-infected skin, suggesting that the DC migration-promoting role of PGE_2_ is independent of IL-1α/β in our model. In DC adoptive transfer experiments, EP2/EP4, but not IL-1R, was needed on the moving DCs for full-fledged migration, supporting different modes of action for PGE_2_ and IL-1α/β. In summary, our data highlight an important role for PGE_2_ in guiding DCs to dLNs in an IL-1–independent manner.

## Key Points

- BCG-triggered PGE_2_ release mobilizes skin DCs to the draining lymph node.
- Migrating DCs use EP2 and EP4 to relocate to the draining lymph node.
- Live BCG bacilli are needed for PGE_2_-mediated DC migration.

## Introduction

Dendritic cells (DCs) in peripheral sites, such as skin, sample microbial Ag from invading pathogens and relocate via lymphatics to the draining lymph node (dLN) to prime T cells (1, 2). Hence DC migration is central for initiating adaptive immunity. Several factors contribute to the complex events that propel the relocation of Ag-bearing DCs from the periphery to dLNs. Sphingosine-1-phosphate (3), Rho GTPases Rac1 and Rac2 (4), and ROCK kinase (5, 6) have previously been shown during both steady-state and inflammation to promote DC cytoskeletal rearrangements that lead to movement. Rear-end, actin myosin–mediated contractions by ROCK kinase are particularly important for movement through interstitium (6). Adhesion molecules such as integrins seem to be dispensable for DC migration during steady state (5), but not inflammation (6–8). In addition, the chemokine receptor CCR7 governs DC chemotaxis under both inflammation and steady state (9). Indeed, the CCR7 ligands CCL19 and CCL21 broadly orchestrate CCR7-directed DC migration: from entry into initial lymphatic vessels in the periphery, egress from collecting vessels at the subcapsular sinus of the LN, to intranodal migration (10, 11).

There is a large body of data on the role for proinflammatory cytokines in regulating DC migration to dLNs (1). Most of these observations come from experiments performed on skin, which houses large populations of DCs and is a readily accessible surface for experimentation in mice. Particular attention has been directed to IL-1α/β. IL-1α is produced in active form and can be both membrane bound and soluble (12), although IL-1β is produced as a procytokine that is activated (cleaved) by caspase-1 (13). Important studies show that a localized injection of IL-1β can trigger egress of DCs from skin and their subsequent accumulation in dLNs (14, 15). Preconditioning the inoculation site in the skin with IL-1α also enhances DC migration from that location to dLNs (16). Similar effects can be reproduced by injecting TNF-α (14–16). IL-1α/β is also known to trigger release of PGE_2_, an inflammatory mediator derived from arachidonic acid that can in itself promote DC migration (17). It is incompletely understood to what extent PGE_2_-mediated DC migration is under control of IL-1.

Substantially less is known regarding DC migration and the active transport of Ag to dLNs in response to microorganisms. In this context, we have been studying the mobilization of DCs from skin to dLN during infection with *Mycobacterium bovis* Bacille Calmette-Guérin (BCG), the live-attenuated vaccine for tuberculosis. Using a CFSE fluorochrome-based assay to track the movement of skin DCs to the dLN (18), we found that the migration of DCs from skin and the accompanying transport of BCG into the dLN is regulated by IL-1R-I and MyD88 (19). That said, IL-1R-I and MyD88 signaling account for only part of this response, and hence many details behind the relocation of DCs to the dLN in our model remain at large. In this study, we reveal an important contribution of the inflammatory lipid mediator PGE_2_, produced early after BCG infection in the skin, in mobilizing skin DCs and BCG into the dLN. The migration-promoting actions of PGE_2_ are independent of IL-1α/β and require viable bacilli.

## Materials and Methods

### Mice

IL-1R-I^−/−^ mice (20) were purchased from Jackson Laboratory (Bar Harbor, ME). IL-1α^−/−^/IL-1β^−/−^ mice (21) were kindly provided by Dr. A. Zychlinsky (Max-Planck-Institut für Infektionsbiologie, Berlin, Germany). C57BL/6NRj mice were purchased from Janvier Labs (Le Genest-Saint-Isle, France) and used as wild-type (WT) controls. Animals were maintained at the Department of Comparative Medicine, Karolinska Institutet. Both male and female mice between 8 and 12 wk old were used. Animals were housed and handled according to the directives and guidelines of the Swedish Board of Agriculture, the Swedish Animal Protection Agency, and Karolinska Institutet. Experiments were approved by the Stockholm North Animal Ethics Council.

### Mycobacteria

*M. bovis* BCG strain Pasteur 1173P2 or BCG Pasteur expressing RFP (22) were expanded in 7H9 broth supplemented with albumin, dextrose, catalase (ADC) (BD Clinical Sciences) as previously described (23). Briefly, mycobacterial stocks were aliquoted in PBS and stored at −80°C until further use. Quantification of stocks and of bacillary load in LNs was performed by determination of CFUs on 7H11 agar supplemented with OADC (BD Clinical Sciences). For generation of heat-inactivated BCG (HI-BCG), mycobacteria prepared as described earlier were autoclaved (121°C, 40 min), cooled to room temperature, and stored at −80°C for future use. HI-BCG did not regrow on 7H11 agar.

### Inoculation of mice

Animals were inoculated in the hind footpad with 1 × 10^6^ CFUs of BCG in 30 μl of PBS. Control animals received 30 μl of PBS only. For CFSE-based assessment of DC migration from the footpad skin to the dLN as previously described (18), BCG- or PBS-inoculated animals were injected 24 h before sacrifice in the same footpad with 20 μl of 0.5 mM CFSE (Sigma). In certain experiments, animals were treated with the cyclooxygenase (COX)-1 and COX-2 inhibitor indomethacin (Sigma). Briefly, animals were injected i.p. with indomethacin at 2 mg/kg in 200 μl. Two hours later, animals were injected in the footpad with BCG as described earlier. Twenty-four hours after BCG, animals received a second injection with indomethacin. For studying the arrival of BCG to the dLN, a single dose of 4 mg/kg indomethacin was administered i.p. 2 h before BCG footpad injection. Where noted, the EP2 (PF04418948) and EP4 (L-161,982) PGE_2_ receptor antagonists (both from Sigma) were injected i.p. daily into mice at 10 mg/kg in 200 μl. For DC adoptive transfer experiments, 1–2 × 10^6^ naive bone marrow–derived DCs (BMDCs) were labeled with 3 μM CFSE and injected in the footpad in 20 μl. Two hours after DC transfer, the same footpads were inoculated with PBS or BCG. For studying gene expression and microsomal PGE synthase-1 (mPGES-1) and PGE_2_ synthesis in the skin, mice were inoculated with BCG in 5 μl of PBS in the ear dermis. An equal volume of PBS was injected as a control.

### Generation of BMDCs

BMDCs were generated by culturing mouse bone marrow cells with recombinant GM-CSF (Invitrogen) for 6 d as previously described (23). Resulting cells were enriched with magnetic selection for CD11c (Miltenyi). In certain experiments, naive CD11c^+^ BMDCs were incubated for 60 min at 37°C with EP2 and EP4 receptor antagonists (10 μM each), washed, and either plated in vitro or inoculated into footpads.

### Generation of single-cell suspensions from tissue

Footpad-draining popliteal LNs (pLNs) were aseptically removed and processed as previously described (19). Briefly, pLNs were transferred to microcentrifuge tubes containing FACS buffer (5 mM EDTA and 2% FBS in PBS) and gently homogenized using a tissue grinder. Resulting single-cell suspensions were counted by Trypan blue exclusion. For CFU determinations, cells suspensions were plated on 7H11 agar as described earlier. For analysis of gene expression by PCR, ears were excised, transferred into TRIzol reagent (Sigma), and homogenized in a TissueLyser with the help of lysis beads (both Qiagen), according to the instructions of the manufacturer. For analysis of PGE_2_ synthesis, ears were excised, transferred into Calibrator diluent buffer, and homogenized in a TissueLyser, and PGE_2_ release was measured by ELISA according to the instructions of the manufacturer (R&D Systems).

### Flow cytometric staining

BMDCs or single-cell suspensions from pLNs were incubated with various combinations of fluorochrome-conjugated anti-mouse mAbs specific for CD11b (M1/70), CD11c (HL3), CD40 (HM40-3), MHC class II I-A/I-E (M5/114.15.2) (BD Biosciences), CD86 (GL-1), CD326/EpCAM (G8.8), and CD103 (2E7) (BioLegend) for 45 min at 4°C in FACS buffer containing 0.5 mg/ml anti-mouse CD16/CD23 (2.4G2) (BD Biosciences), except for CCR7 (4B12) and accompanying isotype control (rat IgG2a) (both from Miltenyi), where incubations were performed for 10 min at room temperature. Flow cytometry was performed on an LSR-II with FACSDiva software (BD Biosciences). Acquired data were analyzed with FlowJo software (BD Biosciences).

### Real-time TaqMan PCR

RNA was extracted from ear homogenates and reverse transcribed into cDNA using Moloney murine leukemia virus reverse transcriptase (Thermo Fisher Scientific). Real-time PCR was performed on an ABI PRISM 7500 sequence detection system (Applied Biosystems) using commercially available primer pairs and TaqMan probes for COX-1, COX-2, mPGES-1, TNF-α, CCR7, ICAM-1, ICAM-2, VCAM-1, and GAPDH (all from Thermo Fisher Scientific). The relative expression of cytokines or inflammatory molecules was determined by the 2^(−△△CT)^ method in which samples were normalized to GAPDH and expressed as fold change over uninfected.

### Confocal microscopy

Ears infected with BCG-RFP were fixed overnight with 4% paraformaldehyde/PBS followed by dehydration in 30% sucrose/PBS before embedding in Tissue-Tek OCT freezing media (Sakura Finetek). A total of 16- to 20-μm-thick sections were cut on a Microm HM 560 cryostat (Thermo Scientific) and adhered to Superfrost Plus slides (VWR). Sections were permeabilized and blocked in PBS containing 0.3% Triton X-100 (Sigma) and 10% goat serum (Jackson Immunoresearch). This was followed by incubation with polyclonal rabbit anti–mPGES-1 (Cayman Chemicals) and staining with Alexa Flour 488–conjugated goat anti-rabbit secondary Ab (Invitrogen). Slides were counterstained with Hoechst and mounted with Prolong Gold (both Invitrogen). 3D image stacks were acquired on a LSM 800-Airy confocal microscope (Carl Zeiss MicroImaging). Images are displayed as 2D maximum intensity projections using Fiji software (ImageJ) (24).

### Statistical analyses

The significance of differences in data group means was analyzed by Student *t* test or ANOVA where appropriate, using GraphPad Prism (GraphPad Software) or JMP (SAS Institute), with a cutoff of *p* < 0.05. In some experiments, outliers were excluded from analysis following Grubbs test for outliers (GraphPad, QuickCalcs).

## Results

### BCG infection in the skin induces expression of COX-2, mPGES-1, and PGE_2_

**FIGURE 1.**
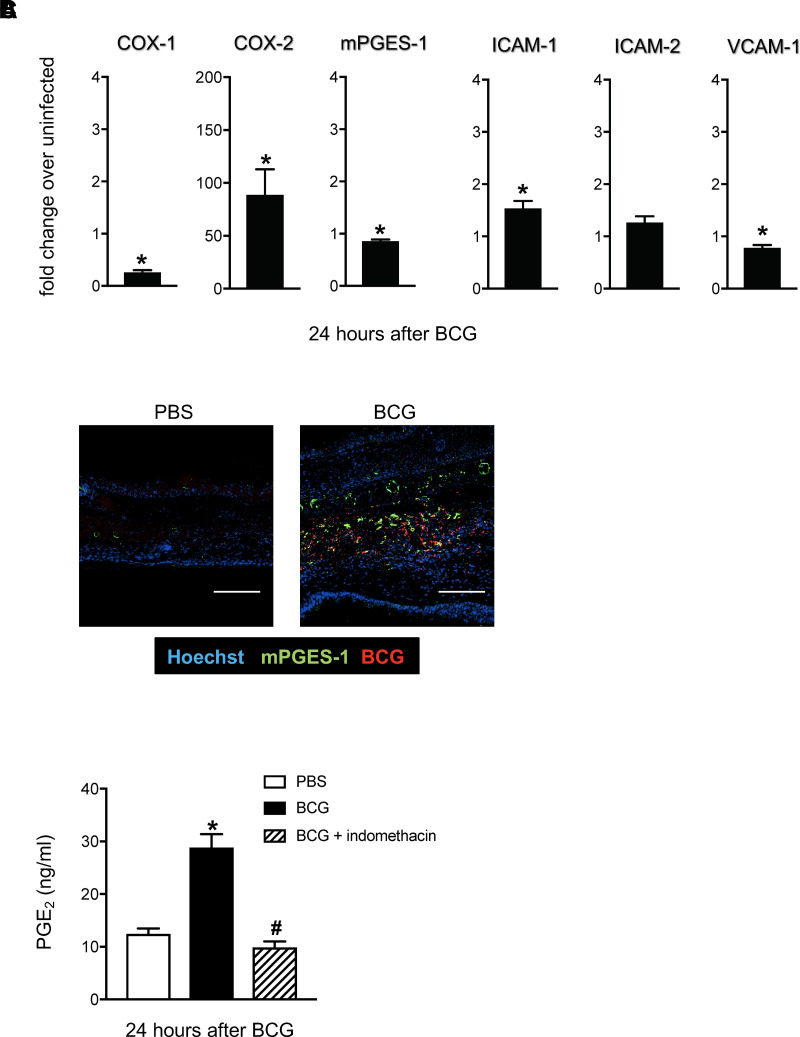
Increased expression of COX-2, mPGES-1, and PGE_2_ in skin after BCG injection. WT mice were inoculated intradermally with BCG in the ear pinna. Control animals were injected with PBS. Ears were removed, processed, and subjected to RNA extraction and cDNA synthesis (**A**), confocal microscopy (**B**), or measurement of PGE_2_ by ELISA (**C**). (A) Expression of COX-1, COX-2, mPGES-1, ICAM-1, ICAM-2, and VCAM-1 mRNA relative to GAPDH was determined by TaqMan PCR. Fold change of BCG-inoculated mice over PBS-injected (uninfected) controls is graphed. Five animals per group. One of at least two independent experiments. (B) Micrograph showing distribution of BCG (red) and mPGES-1 (green) in ears. Nuclei were counterstained with Hoechst (blue). Scale bars (white) represent 100 μm. (C) Mice were treated i.p. with indomethacin 2 h before ear injections. Control-treated animals received DMSO. PGE_2_ levels were determined by ELISA. Three to five mice per group. Bars indicate SEM. Asterisks (*) denote statistically significant difference between BCG-infected and PBS-injected groups. Octothorpe (#) denotes statistically significant difference between BCG-infected and indomethacin-treated, BCG-infected mice.

BCG is a stark inducer of inflammation, but expression of proinflammatory mediators at the site of BCG inoculation in the skin has not been readily investigated, especially at the onset of infection. The lipid mediator PGE_2_ is recognized as an early, active driver of inflammation with a plethora of effects, including regulation of DC responses (17). To investigate the link between BCG and PGE_2_ in skin, we analyzed the expression of PGE_2_ and PGE_2_ synthesis-promoting enzymes COX-1, COX-2, and mPGES-1 in the ear dermis of C57BL/6 WT mice inoculated with BCG at that site. Substantive RNA accumulation of COX-2 was observed in BCG-injected skin 24 h later as measured by TaqMan RT-PCR (Fig. 1A). The constitutive isoform of COX, COX-1, was not upregulated by BCG, which seemed instead to inhibit baseline expression of COX-1 mRNA (Fig. 1A). BCG did not invoke enhanced mRNA accumulation of the adhesion molecules ICAM-1, ICAM-2, or VCAM-1, which are often upregulated during inflammation (Fig. 1A). Surprisingly, mRNA levels of the inflammation-inducible mPGES-1, an enzyme that catalyzes the terminal step of PGE_2_ synthesis (25), were not upregulated by BCG (Fig. 1A). However, imaging of BCG-infected ears revealed substantial expression of mPGES-1 protein proximal to BCG bacilli (Fig. 1B). PGE_2_ synthesis in the skin was elevated 24 h postinfection, corroborating enhanced expression of COX-2 and mPGES-1 at the site of BCG infection (Fig. 1C). Importantly, PGE_2_ synthesis was blocked by in vivo treatment with the COX-1/COX-2 antagonist indomethacin (Fig. 1C). These observations demonstrate that BCG skin infection triggers early expression of COX-2 and mPGES-1 to promote PGE_2_ synthesis.

### COX-derived PGE_2_ controls skin DC migration and BCG entry into dLNs

**FIGURE 2.**
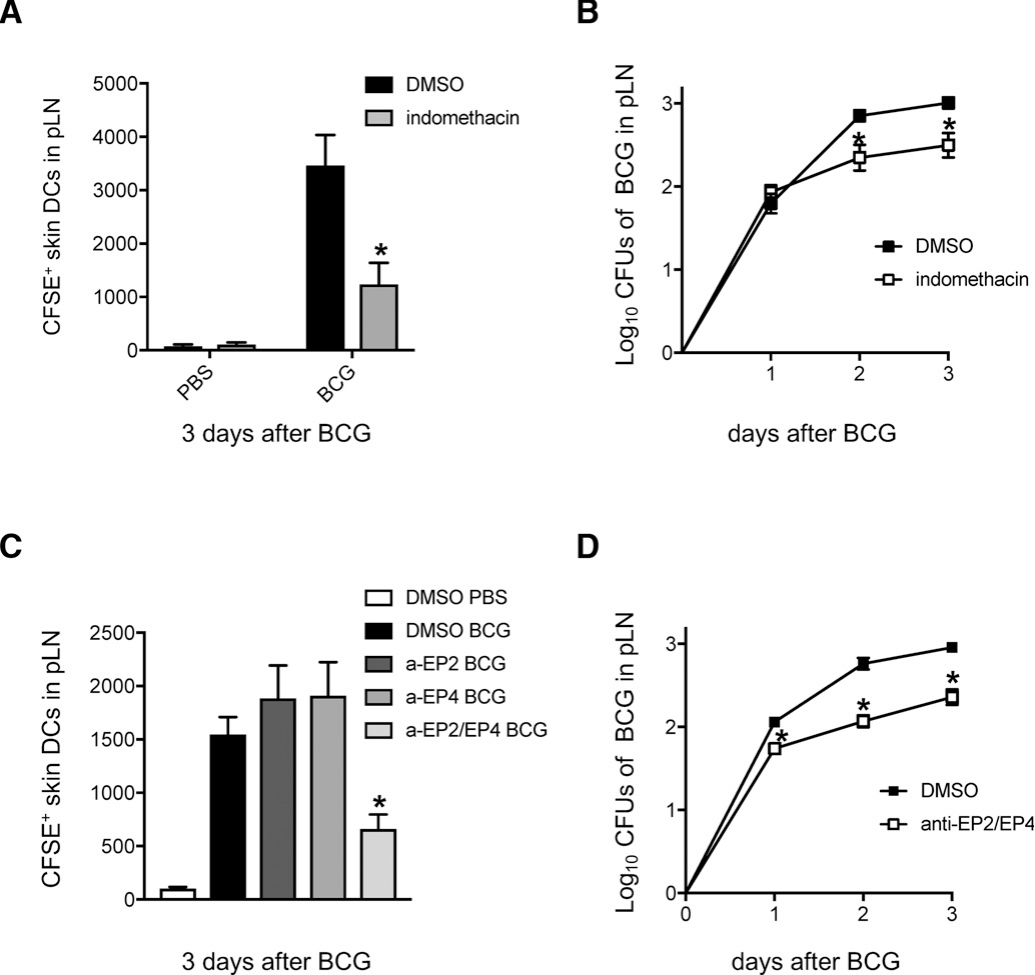
Indomethacin and combined treatment with EP2 and EP4 antagonists mute mobilization of skin DCs and BCG to dLNs. WT mice were treated with indomethacin (**A** and **B**) or with antagonists for EP2, EP4, or both (**C** and **D**), and infected in the footpad with BCG. Total number of CFSE^+^ skin DCs (MHC^high^ CD11c^int^ cells) in BCG-draining pLNs was determined 3 d postinfection (A and C). (B and D) CFUs of BCG in the pLNs of WT mice receiving indomethacin or DMSO control (B) or receiving combined anti (a)-EP2/EP4 treatment or DMSO control (D). Five to 10 animals per time point and group depicted in BCG-infected cohorts, and five animals for PBS-injected DMSO controls. Bars indicate SEM. Asterisks (*) denote statistically significant difference between BCG-infected, indomethacin-treated mice and BCG-infected DMSO controls (A and B), or BCG-infected anti-EP2/EP4–treated mice and BCG-infected DMSO controls (C and D). CFSE^+^ skin DCs reported in BCG-infected groups (A and C) are statistically significant compared with uninfected controls.

Next, we addressed the impact of COX-derived PGE_2_ in skin DC migration. To this end, animals were treated with indomethacin, and skin DC migration to dLNs was investigated using our CFSE-based migration assay (18). Due to methodological impediments, the ear is not amenable to this assay. Instead, infection is performed in the footpad skin to quantify relocation of skin DCs from that site to the dLN (the pLN). Prior results from the combined use of these models support the detection of TNF-α and IL-1α/β in infected skin with a role for TNFR-I and IL-1R-I in skin DC migration to dLNs (19, 26). Indomethacin treatment had a visible, negative impact on BCG-mediated skin DC migration to dLNs (Fig. 2A). In line with muted skin DC migration, BCG levels in the dLN were also reduced in indomethacin-treated mice (Fig. 2B). In agreement with the earlier effects of indomethacin, relocation of skin DCs and BCG to dLNs was also reduced in mice receiving combined treatment with antagonists for the PGE_2_ receptors EP2 and EP4 (Fig. 2C, 2D). Collectively, these observations point to a central role for COX and the PGE_2_ receptors EP2 and EP4 in regulating skin DC mobilization and BCG entry into dLNs.

### Live BCG is required for COX-mediated skin DC migration

**FIGURE 3.**
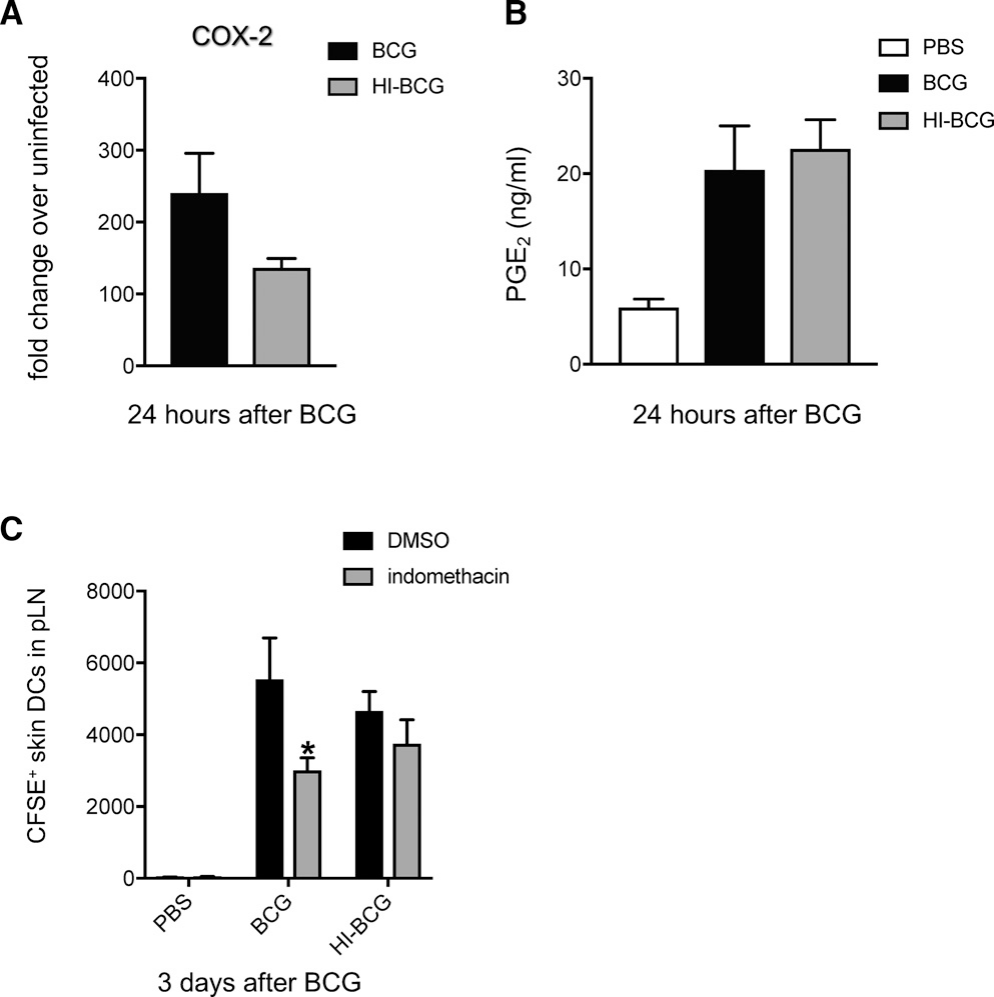
HI-BCG induces skin DC migration independent of COX. Mice were injected in the ear pinna with BCG and HI-BCG (**A** and **B**). One day later, ears were removed, processed, and subjected to RT-PCR for COX-2 (A) or measurement of PGE_2_ by ELISA (B). (**C**) Mice were treated i.p. with indomethacin or DMSO control and infected with BCG or HI-BCG in the footpad. Three days postinfection, pLNs were excised, processed, and analyzed by flow cytometry. The total numbers of CFSE^+^ skin DCs (MHC^high^ CD11c^int^ cells) in pLNs are shown. Five to seven animals per group. One of at least two independent experiments is shown. Bars indicate SEM. Asterisk (*) denotes statistically significant difference between BCG-infected, indomethacin-treated mice and BCG-infected, DMSO controls. COX-2 transcripts, PGE_2_ levels, and CFSE^+^ skin DCs reported in BCG and HI-BCG groups are statistically significant compared with PBS.

Because BCG is given as a live vaccine, the importance of bacillary viability in promoting DC migration through the COX-PGE_2_ axis was investigated. BCG was inactivated by heat (autoclaving) and used first to gauge expression of COX-2 and PGE_2_ in skin. Interestingly, HI-BCG also prompted COX-2 mRNA accumulation (Fig. 3A). Despite a tendency for lower transcript levels compared with live BCG, the difference was not statistically significant or reflective of an actual difference in skin PGE_2_ production between BCG and HI-BCG (Fig. 3B). Similarly, skin DC migration initiated by HI-BCG was similar to that initiated by BCG (Fig. 3C). However, skin DC migration triggered by HI-BCG was resistant to indomethacin treatment (Fig. 3C). Thus, although dead mycobacteria suffice to shuffle skin DCs to dLNs, the COX-PGE_2_ pathway of skin DC migration seems to be preferentially deployed by active BCG bacilli.

### COX-2 and PGE_2_ expression during BCG skin infection are independent of IL-1α/β

**FIGURE 4.**
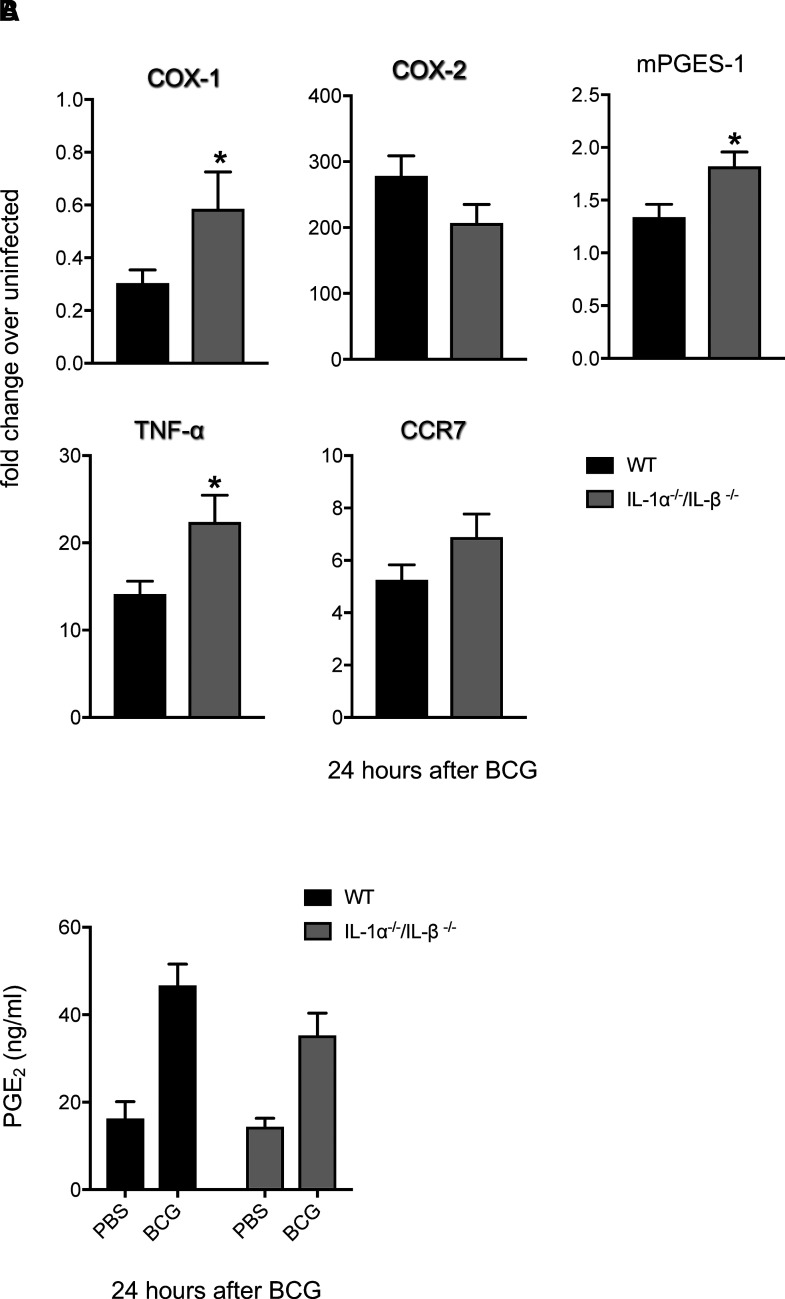
Expression of COX-1, COX-2, mPGES-1, TNF-α, CCR7, and PGE_2_ in the skin of BCG-infected WT and IL-1α^−/−^/IL-1β^−/−^ mice. WT and IL-1α^−/−^/IL-1β^/−^ mice (**A** and **B**) were injected in the ear pinna with BCG. Control animals received PBS. One day later, ears were removed and subjected to RNA extraction and cDNA synthesis. Expression of COX-1, COX-2, mPGES-1, TNF-α, and CCR7 transcripts relative to GAPDH was determined by TaqMan PCR (A). Fold change of BCG-inoculated mice over PBS-injected controls graphed. Data were pooled from two independent experiments, totaling 6–10 animals per group. Levels of PGE_2_ in ears of WT and IL-1α^−/−^/IL-1β^−/−^ mice were measured by ELISA (B). Four to five animals per group. One of three independent experiments. Bars indicate SEM. Asterisks (*) denote statistically significant difference between WT and IL-1α^−/−^/IL-1β^−/−^ cohorts. For both WT and IL-1α^−/−^/IL-1β^−/−^ groups, measured transcripts (A) and PGE_2_ levels (B) in BCG-infected mice are statistically significant compared with PBS.

We have previously shown that IL-1R-I^−/−^ mice have an impaired ability to mobilize skin DCs to dLNs in response to BCG skin infection (19). To explore whether the COX-PGE_2_ pathway is under IL-1α/β control in our model, we infected IL-1α^−/−^/IL-1β^−/−^ mice with BCG in the skin and analyzed transcription of COX-1, COX-2, and mPGES-1. As in WT controls, early, infection-induced mRNA accumulation of COX-2 was enhanced in IL-1α^−/−^/IL-1β^−/−^ mice (Fig. 4A). Expression of TNF-α and CCR7 mRNA was also not reduced by removal of IL-1α/β (Fig. 4A). Albeit the differences were small, infection-induced transcripts for COX-1, mPGES-1, and TNF-α appeared to be somewhat higher in the absence of IL-1α/β (Fig. 4A). In line with intact upregulation of COX-2 mRNA in IL-1α^−/−^/IL-1α^−/−^ mice, BCG-triggered production of PGE_2_ in skin was also intact in the absence of IL-1α/β (Fig. 4B). As such, although both IL-1 and PGE_2_ are important for skin DC migration in our model, these inflammatory mediators represent independent pathways for control of DC relocation to dLNs.

### Requirement for PGE_2_, but not IL-1R, for DC migration is intrinsic to the moving DCs

**FIGURE 5.**
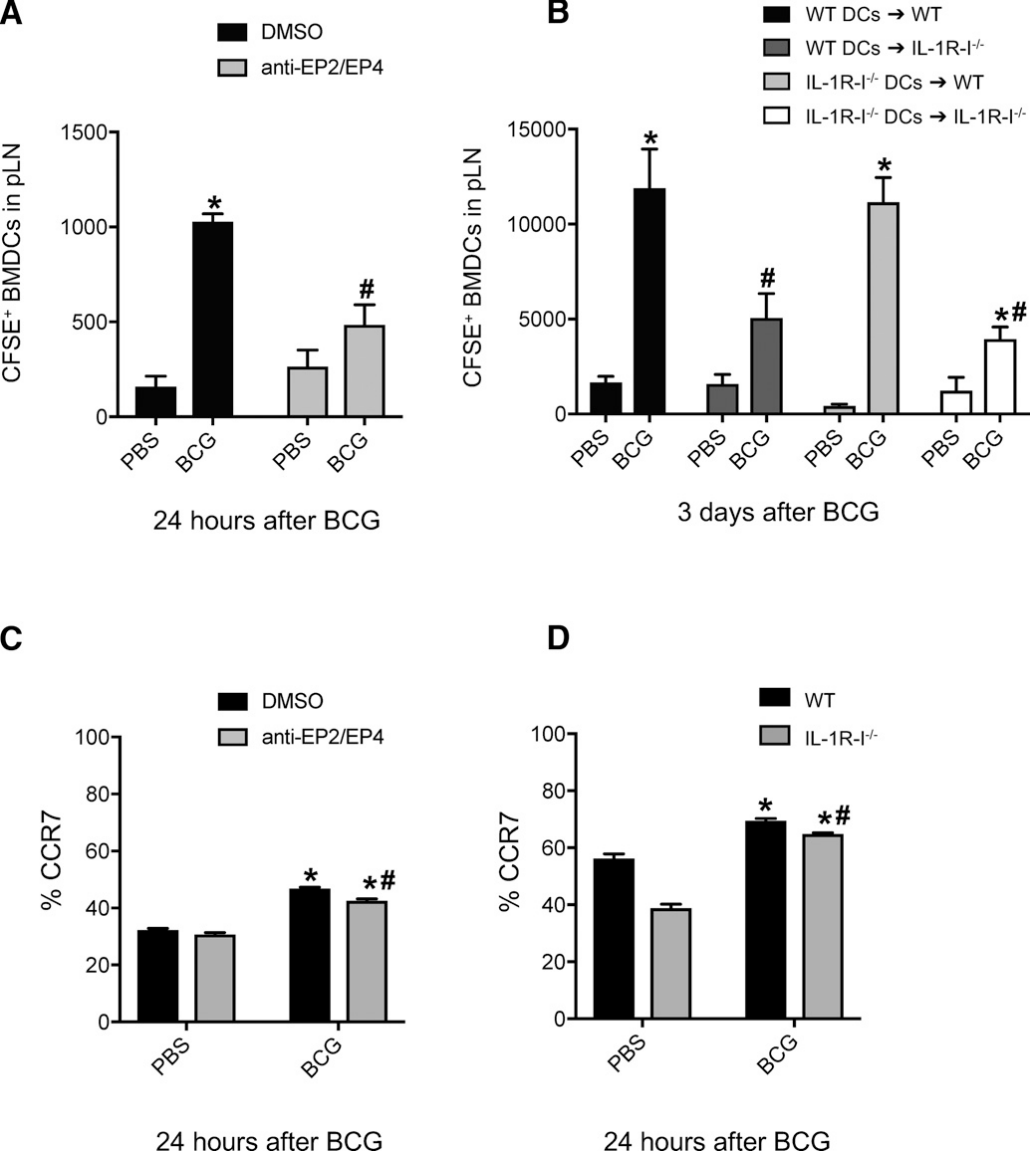
BCG-triggered migration of adoptively transferred BMDCs requires EP2/EP4 receptors, but not IL-1R-I, on the moving DCs. (**A** and **C**) WT BMDCs were labeled with CFSE and treated in vitro with EP2 and EP4 antagonists. Control BMDCs were incubated with DMSO. BMDCs were then injected into the footpad of naive recipients and 2 h later infected with BCG in the same footpad. (**B** and **D**) BMDCs were generated from WT and IL-1R-I^−/−^ mice, CFSE labeled, and injected in the footpad of naive WT or IL-1R-I^−/−^ recipients, respectively. Recipients were then infected 2 h later with BCG in the same footpad. The number of labeled BMDCs reaching the draining pLN was determined by flow cytometry 24 h (A) or 3 d (B) after BCG infection. Migration was measured after 24 h in (A) to avoid potential impact of PGE_2_ receptor recycling after EP2/EP4 blockade. Three to five animals per group. One of three independent experiments is shown. Bars indicate SEM. Asterisks (*) denote statistically significant difference between BCG and PBS groups; octothorpes (#) denote statistically significant difference between anti-EP2/EP4–treated animals and DMSO controls (A and C), between experimental transfers and WT→WT controls (B), or between WT and IL-1R-I^−/−^ DCs (D).

Next, we performed DC adoptive transfers to investigate whether the signals generated by PGE_2_ and IL-1 were needed on the moving DCs. Naive BMDCs from WT mice were treated in vitro with DMSO or EP2 and EP4 antagonists and transferred into the footpad of naive WT recipient animals. The number of BMDCs reaching the dLNs in response to BCG infection in the same footpad was then determined by flow cytometry. Interestingly, BMDCs treated with the EP2/EP4 antagonists did not migrate properly from skin to dLNs in response to BCG (Fig. 5A). This implies that a functional response to PGE_2_ (from these receptors) is needed on the moving DCs for full-fledged, BCG-elicited migration of transferred DCs.

On the contrary, IL-1R-I^−/−^ DCs transferred into WT hosts relocated to the dLN to the same extent as WT DCs (Fig. 5B), suggesting that IL-1R signaling is not needed on the moving DCs. Still, WT DCs transferred into IL-1R-I^−/−^ recipients were muted in their ability to reach the dLN (Fig. 5B). In addition to corroborating the importance of IL-1R signaling for full-fledged BCG-triggered DC migration (19), this observation places the requirement for IL-1R extrinsic to the moving DCs. In this regard, the inability of WT DCs to properly relocate to dLNs in IL-1R-I^−/−^ recipients, but also the inability of EP2/EP4-blocked WT DCs to properly relocate in WT recipients, is not linked to downregulation of CCR7 on the transferred DCs. Indeed, WT, IL-1R-I^−/−^, and EP2/EP4-blocked WT DCs all expressed high surface levels of CCR7 (Fig. 5C, 5D). Furthermore, EP2/EP4-antagonized WT BMDCs, as well as IL-1R-I^−/−^ BMDCs, were fully capable of upregulating costimulatory molecules in response to BCG stimulation in vitro (Supplemental Fig. 1). Hence BMDC activation in response to BCG was intact despite inhibition of PGE_2_ and IL-1R signaling, respectively, on these cells. Altogether, these results show that IL-1R signaling is dispensable on the moving DCs, whereas the migration-promoting effects of PGE_2_ are not.

## Discussion

The mobilization of Ag-laden DCs from the infection site in the periphery to the dLN is needed for T cell priming. Support for this comes from observations, including our own, showing that failure or inhibition of skin DC migration from skin to dLN mutes the expansion of Ag-specific T cells in the dLN (19, 26–28). The movement of DCs from tissue to LN is complex. In the context of infection, many of these details remain unclear. We show in a mouse model that infection with BCG in the skin initiates DC relocation from skin to dLN in a process that relies on COX-derived synthesis of PGE_2_. This is, to our knowledge, the first report demonstrating a role for PGE_2_ in mycobacterial-triggered DC migration. IL-1R signaling has been previously shown to play an important role in mobilizing skin DCs to dLNs in this model (19). We demonstrate, however, that signaling through PGE_2_ represents an IL-1α/β–independent axis of migration triggered by infectious BCG bacilli.

PGE_2_ can orchestrate DC migration in multiple ways. For instance, PGE_2_ dissolves podosomes on DCs, a cytoskeletal rearrangement that leads to migration (29). PGE_2_ has also been shown to both upregulate CCR7 on DCs (30, 31) and to oligomerize CCR7 on DCs (32), which enforces migration. In agreement, DC maturation cocktails containing PGE_2_ generate DCs with superior migratory capacity toward CCR7 ligands (30, 31, 33). In contrast, the presence of PGE_2_ in such cocktails may impair other DC functions. In particular, PGE_2_ may limit cytokine production (30, 33) and in a dose-dependent manner reduce cross-presentation (34). High levels of PGE_2_ stimulation may even suppress DC migration (35).

Another mechanism by which PGE_2_ can facilitate migration is through expression of tissue-degrading matrix metalloproteinase 9 (MMP9) (31). Indeed, MMP9 is used by DCs to migrate out of skin explants (36). In DC adoptive transfer experiments, MMP9 is needed on the moving DCs for it to reach the dLN (31). In our own adoptive transfer experiments, PGE_2_ was needed on the moving BMDCs for full-fledged relocation to dLNs. We observed abundant CCR7 expression on BMDCs, in line with the ability of these DCs to migrate in vitro in response to CCL19, even without receiving microbial activation (19). Furthermore, EP2/EP4 antagonism did not abrogate surface expression of CCR7 on BMDCs. Thus, in our adoptive transfers, PGE_2_-intrinsic effects on DC migration are not due to downregulation of CCR7 on DCs. That said, we cannot rule out the possible contribution of PGE_2_-triggered oligomerization of CCR7 on DCs (32).

High levels of CCR7 also decorated the surface of IL-1R-I^−/−^ BMDCs. Unlike EP2/EP4 signaling, IL-1R was not needed on the moving DCs for maximized relocation to dLNs. It was needed only on the recipient. This observation is different from the requirement for MyD88 in our DC adoptive transfer model, which unlike IL-1R is needed both intrinsic and extrinsic to the moving DCs (19). Because MyD88 also orchestrates TLR signaling in response to mycobacteria, we speculate that the intrinsic requirement for MyD88 is coupled to TLR signaling on the moving DCs, while IL-1–derived signals originate from the environment as a consequence of BCG infection. Important prior work on the role of IL-1α/β during *M. tuberculosis* infection shows that it confers host resistance through production of PGE_2_ (37). During pulmonary infection with *M. tuberculosis*, IL-1–triggered PGE_2_ contributes to mycobacterial containment by countering IFN-α/β–mediated pathology (37). IL-1 is also important for host control of BCG infection (38). Our studies that focus on the much earlier DC migratory response to mycobacteria reveal independent actions for IL-1 and PGE_2_ in mobilizing skin DCs to dLNs. This difference could be because of the large difference in virulence between the mycobacteria used, timeline, and/or tissue type investigated.

Surface expression of all four murine PGE_2_ receptors (EP1–4) has been demonstrated on DCs (39). Although EP1 and EP3 signaling predominates during DC differentiation (40), EP2 and EP4 may be more important during infection because both are further upregulated in response to microbial stimulation (39). Indeed, EP2^−/−^ mice display enhanced susceptibility to *M. tuberculosis* (41). Given the earlier data, we focused our investigations on EP2 and EP4, and show using EP2 and EP4 antagonists that both of these receptors are needed for efficient skin DC migration and BCG influx to dLNs in our model. EP4 has been shown to mediate relocation of Langerhans cells to dLNs as measured by FITC-skin painting (42). Both EP2 and EP4 are needed for adoptively transferred DCs to reach dLNs in an MMP9-dependent manner (31). Combined signaling between EP2 and EP4 has also been demonstrated in migration of human monocyte-derived DCs across transwells (33, 43). We did not study the contribution of EP1 or EP3 in DC migration and therefore cannot formally exclude a role for either receptor in the latter process. Still, our results with BCG are clearly supported by the aforementioned work that recognizes the importance of EP2 and EP4 in DC migration, as well as the joint effort conferred by these receptors in promoting DC migration to dLNs.

BCG is given clinically as a live vaccine in the skin. Seminal work done in the late 1950s demonstrated that inactivating BCG markedly reduced its ability to protect guinea pigs from lethal challenge with *M. tuberculosis* (44, 45). Intravesical instillation with live BCG is also reported to be superior to HI-BCG in the immunotherapy of bladder cancer (46). A proposed advantage of live BCG is that it seems to promote T cell responses against secreted mycobacterial Ags (47). Interestingly, inactivated BCG seems to remain at the site of injection in the skin, while live bacilli move to dLNs (48). That said, skin DC migration was not investigated in the latter study. Our results show that inactivating BCG by steam sterilization does not interfere with the ability of the bacilli to mobilize skin DCs to the dLNs. The capacity to trigger DC migration in the absence of viable bacilli could be beneficial in certain immunization protocols, such as ongoing clinical efforts to introduce whole mycobacterial cell lysate preparations as boosters or prophylactic vaccines against *M. tuberculosis* (49).

Although inactivated BCG can mobilize skin DCs to dLN, metabolically active BCG are needed to drive the COX-derived PGE_2_ mechanism of DC migration. HI-BCG is structurally compromised and lacks enzymatic function, both of which are needed to consummate infection. BCG-infected DCs have been shown to display arrested movement compared with uninfected DCs (50, 51). We confirm that a large number of skin DCs that relocate to dLNs in response to BCG are not found carrying live BCG bacilli (19). Mobilizing infected DCs to dLN may thus be under stricter control to prevent bacterial dissemination through lymphatics. In contrast, the presence of live mycobacteria in dLN coincides with priming of protective CD4^+^ T cells at the same site (19, 52–54). We speculate that infection-mediated cytoskeletal rearrangements or cell morphological changes render DCs amiable to the migration-promoting actions of PGE_2_. This could prompt in turn initial detachment of DCs from matrix. Alternatively, during live infection, a larger source of PGE_2_ could be derived from infected DCs to create a local microenvironment rich in PGE_2_ to support DC displacement to dLN. More work is needed to unravel how live bacilli invoke COX-derived PGE_2_ release, a pathway that could be manipulated to improve priming by increasing DC transport of live bacilli to dLNs.

In summary, results from our model unfold important, independent contributions by IL-1 and PGE_2_ in guiding DC traffic to dLNs in response to BCG skin infection. The migration-promoting actions of COX-derived PGE_2_ required stimulation with live BCG bacilli and were intrinsic to the moving DCs in a DC adoptive transfer setting. These findings that unfold early events after BCG stimulation in vivo may lead to strategies targeting PGE_2_ receptor signaling to boost skin DC migration to dLNs.

## Supplementary Material

Data Supplement
