# Supplementary material for: Cyclooxygenase-Derived Prostaglandin E2 Drives IL-1–Independent Mycobacterium bovis Bacille Calmette-Guérin–Triggered Skin Dendritic Cell Migration to Draining Lymph Node
Source: J Immunol. 2022 Jun 1;208(11):2549–57. doi: 10.4049/jimmunol.2100981 (PMC9161203; doi:10.4049/jimmunol.2100981)
Supplement: Data Supplement [file JI_2100981.zip › JI_2100981_Supplemental_1.pdf]

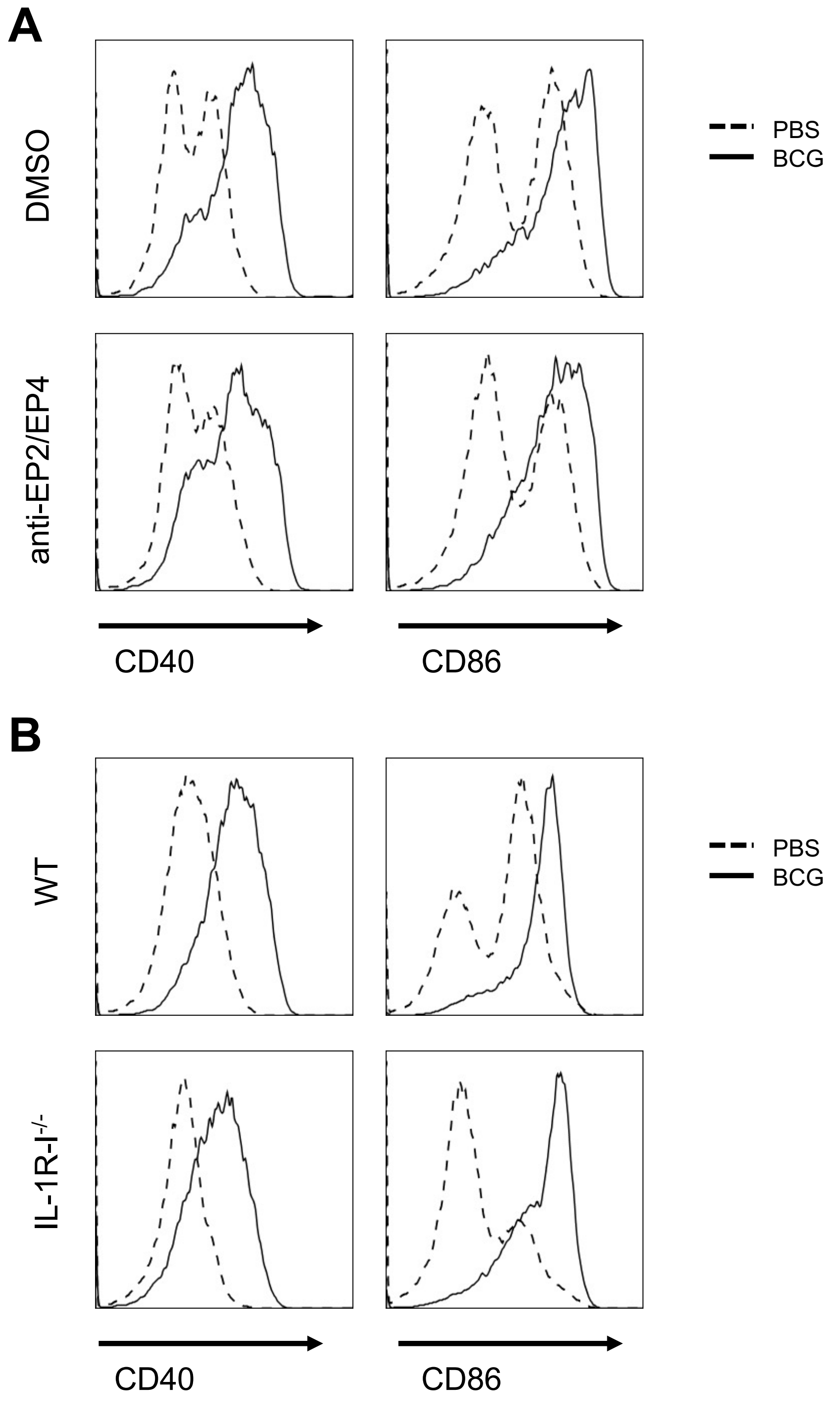

SUPPLEMENTARY FIGURE 1. Surface expression co-stimulatory molecules in naïve and BCG-stimulated BMDCs. (A) BMDCs were generated from WT mice and treated *in vitro* with DMSO or EP2/EP4 antagonists for one hour, washed, and stimulated overnight with PBS or BCG at a multiplicity of infection (MOI) of 1. (B) WT and IL-1R-I$^{-/-}$ BMDCs were left untreated or infected overnight with BCG at a MOI of 1. (A and B) DCs were thereafter stained for CD40 and CD86 and analyzed by flow cytometry. Histograms showing up-regulation of CD40 and CD86 on uninfected (dashed lines) and BCG-stimulated BMDCs (solid lines). One of 2 independent experiments.
